# Physical Function Changes in Older Adults Living in Temporary Housing after the Great East Japan Earthquake

**DOI:** 10.31662/jmaj.2025-0121

**Published:** 2025-07-02

**Authors:** Toshiki Abe, Hiroaki Saito, Nobuaki Moriyama, Michio Murakami, Naomi Ito, Yoshitaka Nishikawa, Morihito Takita, Isamu Amir, Yoshitaka Shiba, Takeaki Ishii, Sae Ochi, Chika Yamamoto, Tianchen Zhao, Makoto Kosaka, Masaharu Tsubokura

**Affiliations:** 1 Department of Radiation Health Management, Fukushima Medical University School of Medicine, Fukushima, Japan; 2 Department of Internal Medicine, Soma Central Hospital, Fukushima, Japan; 3 National Institutes of Biomedical Innovation, Health and Nutrition, Osaka, Japan; 4 Department of Public Health, Fukushima Medical University School of Medicine, Fukushima, Japan; 5 Center for Infectious Disease Education and Research, The University of Osaka, Osaka, Japan; 6 Department of Health Informatics, Kyoto University School of Public Health, Kyoto, Japan; 7 Research Division, Medical Governance Research Institute, Tokyo, Japan; 8 Department of Physical Therapy, Fukushima Medical University School of Health Sciences, Fukushima, Japan; 9 Nippon Boehringer Ingelheim Co., Ltd., Tokyo, Japan; 10 Department of Laboratory Medicine, The Jikei University School of Medicine, Tokyo, Japan

**Keywords:** physical function, balance function, post-disaster, temporary housing, Great East Japan Earthquake

## Abstract

**Introduction:**

The aging of the global population presents significant challenges in extending healthy life expectancy, particularly among older adults. The prevalence of musculoskeletal disorders and the growing demand for nursing care are expected to reduce healthy life expectancy. These outcomes are particularly influenced by disasters that cause major environmental changes. This study aimed to clarify the long-term effects on physical function resulting from temporary changes in the living environment of older adults who relocated to temporary housing (TH) following the Great East Japan Earthquake.

**Methods:**

This retrospective cohort study included 4,680 residents of Soma City, Fukushima Prefecture, aged ≥64 years, who underwent locomotor function examinations between 2013 and 2022. Participants were categorized into two groups: TH (n = 440) and control (n = 4,240). The primary outcomes were grip strength and one-leg standing time. A growth curve model was used to estimate the longitudinal associations.

**Results:**

The mean age- and sex-adjusted one-leg standing time was lower in the TH group than in the control group in 2013 (35.5 seconds vs. 39.3 seconds) and remained lower thereafter. The results showed an association between one-leg standing time and TH experience (intercept estimate: −4.32, 95% confidence interval: −7.49 to −1.16). No differences in grip strength were observed between the groups.

**Conclusions:**

These results suggest that long-term support is necessary for evacuees, and it is essential to develop and implement support measures that effectively address muscle weakness and other aspects of physical function.

## Introduction

Maintaining musculoskeletal function is crucial for healthy life expectancy, particularly among older individuals ^(1)^. Japan has one of the highest aging rates in the world, with an aging rate of 28.6% in 2022 ^(2)^. Since healthy life expectancy refers to the period during which a person can live a long and healthy life, extending it is important for older individuals. However, various factors can shorten this period. Diseases and disorders of the locomotor system, reduced balance ability, and decreased muscle strength are believed to shorten healthy life expectancy ^(3), (4)^. Musculoskeletal disorders are strongly associated with falls, which necessitate higher levels of care ^(5)^, reduce independence and healthy life expectancy, and negatively impact overall quality of life ^(6), (7)^. Therefore, identifying factors associated with such disorders is of utmost importance.

Older adults are particularly vulnerable to changes in their living environment and the development of musculoskeletal disorders following a disaster ^(8), (9)^. Care for older adults after a disaster is an urgent issue, particularly in areas with aging populations. Living in shelters can decrease physical activity and muscle strength, increasing the risk of fractures and frailty due to falls, even among healthy older adults ^(8), (10)^. Physical function assessments, such as grip strength and standing balance measurements, are important for understanding these risks ^(11), (12)^. However, little is known about the long-term trends in physical activity among individuals who have experienced even a single change in their living environment.

The Great East Japan Earthquake of March 2011 was a complex disaster that caused various secondary health effects due to evacuation. In Fukushima Prefecture, 121,809 houses were damaged, and many residents lost their homes and relocated to temporary housing (TH) ^(13)^. The evacuation caused secondary health effects in evacuated residents due to changes in lifestyle and social environment ^(14), (15)^. In particular, the impact of evacuations on the level of independence among older adults is an important consideration. The risk of needing care is higher for residents who were forced to change their housing due to the tsunami and earthquake ^(8), (16)^. However, few reports have described ongoing assessments of physical function among residents who were forced to evacuate for extended periods, hindering the identification of factors contributing to the decline in physical function that increases the risk of requiring long-term care. To address these issues, physical function monitoring of residents affected by the disaster was conducted in Soma City, in northeastern Fukushima Prefecture ^(17), (18)^. Although this area was outside the evacuation zone established due to the nuclear disaster, houses in this area were severely damaged by the tsunami. A previous study conducted in Soma City showed that the physical performance of older residents a year after moving to TH was lower than that of those who did not relocate ^(19)^. However, 13 years after the earthquake, the medium- and long-term effects of evacuation on resident’s physical function remain largely unknown.

Therefore, this study aims to clarify the long-term changes in the physical function of residents who moved into TH after the Great East Japan Earthquake. Understanding the long-term effects on evacuees’ physical function after a disaster could provide critical insights to guide long-term support and countermeasures following large-scale disasters.

## Materials and Methods

### Study setting and participants

This retrospective cohort study included residents of Soma City, Fukushima Prefecture, who underwent medical checkups with physical performance assessments between 2013 and 2022. A physical performance assessment program was initiated in September 2012 to monitor the health of individuals who had relocated to TH in Soma City after the Great East Japan Earthquake of 2011 ^(19)^. From 2013 onward, the program was extended to include both individuals who had lived in TH at least once and those who had never lived in TH, as part of specified health checkups or screenings for older adults. The assessments targeted individuals aged ≥64 years, with annual checkups recommended. Recruitment was conducted through public announcements in city publications and other media, providing details about the purpose, dates, locations, and methods of the performance tests. These assessments were carried out annually between September and October. This study utilized data from a physical performance assessment database maintained by Soma City.

### Inclusion and exclusion criteria

The study included individuals aged ≥64 years at the time of examination who underwent at least one performance assessment between 2013 and 2022. Participants with unknown sex or age were excluded from the analysis.

### TH experience group

Participants were divided into two groups: those who had lived in TH at least once (TH experience group: TH group) and those who had never lived in TH (control group). Most residents of TH in Soma City had lived in coastal areas before the disaster and lost their homes due to the tsunami and earthquake. TH refers to structures built on vacant land, not exceeding 29.7 square meters, and funded by public expenditure. These dwellings are primarily single-story buildings equipped with essential amenities such as toilets, baths, and living areas ^(20)^. The TH program began in May 2011. Eligibility for TH was not determined by individual factors such as income or the presence of chronic diseases but rather accommodated those who had lost their homes due to the disaster. As of December 2011, 2,743 people resided in TH in Soma City ^(21)^. This number gradually decreased, and the TH program concluded in March 2017 ^(22)^.

### Outcome

The analysis was based on the results of grip strength and one-leg standing time (OLST) measurements obtained during the physical performance assessment.

### Grip strength measurement

Grip strength was measured using a Grip Strength Tester (ST3, TOEI LIGHT, Saitama, Japan). Participants underwent measurements once on each side while standing, with the higher value recorded ^(23)^. The measurement range was 0-100 kg, with values recorded in 1 kg increments. Grip strength is widely used as an indicator of nutritional status and a predictor of physical function outcomes ^(23)^.

### OLST measurement

The OLST was measured once using the dominant leg with the eyes open. In the OLST, participants self-selected the leg on which they felt more stable and comfortable standing. The OLST was defined as the duration from the moment the leg was raised until it was placed on the floor, touched the opposite leg, or the hand was removed from the hip ^(24)^. The measurement range was 0-60 seconds, recorded in 1-second increments ^(25)^. The OLST is primarily used to assess balance ability ^(26), (27)^ and is associated with falls, fall-related fractures, sarcopenia, and frailty ^(28), (29)^.

### Analysis methods

**Figure 1.**
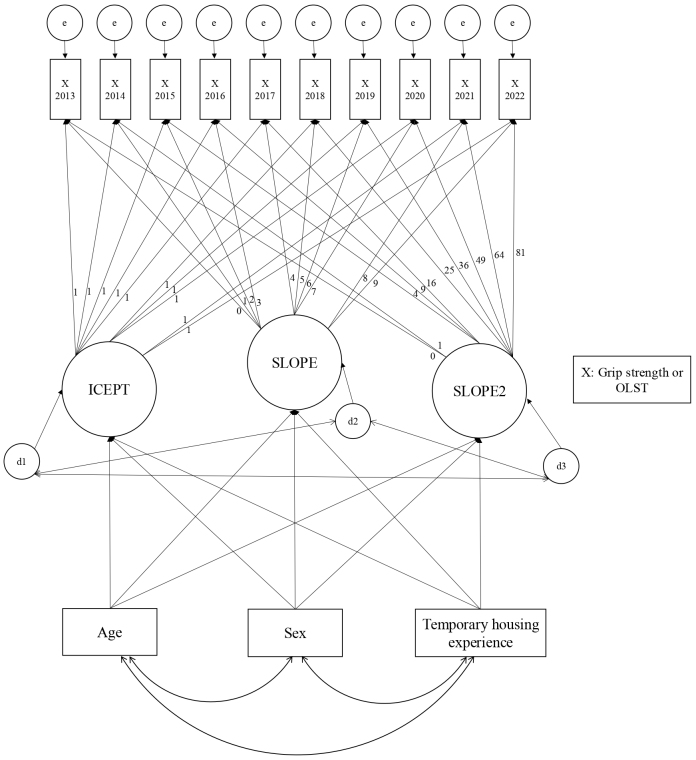
Diagram of the latent growth curve model. Figure 1 shows a latent growth curve model that analyzes the associations among the factors “age,” “sex,” and “temporary housing experience” and their influence on grip strength and one-leg standing time (OLST) results over 10 years (2013-2022). X represents either grip strength or one-leg standing time, both of which were incorporated into the model. The values shown are unstandardized path coefficients.

First, the mean outcome value for each year from 2013 to 2022 was calculated. To address censored data (both left- and right-censoring), we used the Bayesian estimation method. Left-censoring occurred when participants could not maintain the one-leg standing position long enough to record a precise measurement (recorded as “0” but treated as between 0 and 1 s in the analysis). Right-censoring occurred when participants maintained the position for the full testing period (40 s), at which point the test was terminated per standard protocol. The analysis of grip strength was handled in the same manner as the OLST. These mean values were adjusted for sex and age based on the 2015 Japanese population model. Second, to account for the nature of panel data, we constructed a growth curve model (GCM) with grip strength and OLST as dependent variables. To analyze the associations among age, sex, and TH experience over the 10 years (Figure 1), we evaluated four different models for age: continuous linear, dichotomized linear, continuous quadratic, and dichotomized quadratic forms. Based on existing research ^(30)^, we included quadratic terms to account for potential non-linear changes in physical function with age. The linear models are presented in Supplementary Figure 1. Sex (reference = female), age (measured in 2013), and TH experience (TH or control groups) were included as independent variables. Figure 1 illustrates the quadratic latent GCM used to analyze the associations among age, sex, and TH experience and their influence on grip strength and OLST over the 10 years. The model incorporates three latent growth parameters: initial level (ICEPT) representing baseline values in 2013, linear change rate over time (SLOPE), and quadratic or accelerated change over time (SLOPE2). The factor loadings from ICEPT to all observed variables were fixed at 1, while the loadings from SLOPE followed a linear sequence (0, 1, 2, 3, 4, 5, 6, 7, 8, 9), representing years since baseline. The SLOPE2 parameter’s loadings followed quadratic values (0, 1, 4, 9, 16, 25, 36, 49, 64, 81), representing squared time scores. Error terms for each observed variable and disturbance terms for latent variables were included to account for unexplained variance. The analyses were conducted using the Full Information Maximum Likelihood method to account for missing data. Model fit was assessed using the comparative fit index (CFI), root mean square error of approximation (RMSEA), and Akaike information criterion (AIC). A satisfactory fit was defined by CFI ≥ 0.90 and RMSEA < 0.08 ^(31), (32)^. In addition, a smaller AIC value indicates a better fit and a more simplified model ^(31), (32)^. Based on the goodness-of-fit indices shown in Supplementary Table 1 for the age variable, we adopted the continuous quadratic form of age, which demonstrated the best fit among the four models tested. All analyses were conducted using SPSS Amos ver. 28 (IBM Corp., Armonk, NY, USA) and Stata/BE (version 18.0; Stata Corp. LP, College Station, TX, USA), with *p*-values < 0.05 indicating statistical significance.

### Ethics

This study was approved by the Fukushima Medical University Ethics Committee (reference number: REC2024-059). Patient consent was obtained on an opt-out basis.

## Results

### Participant characteristics

**Figure 2.**
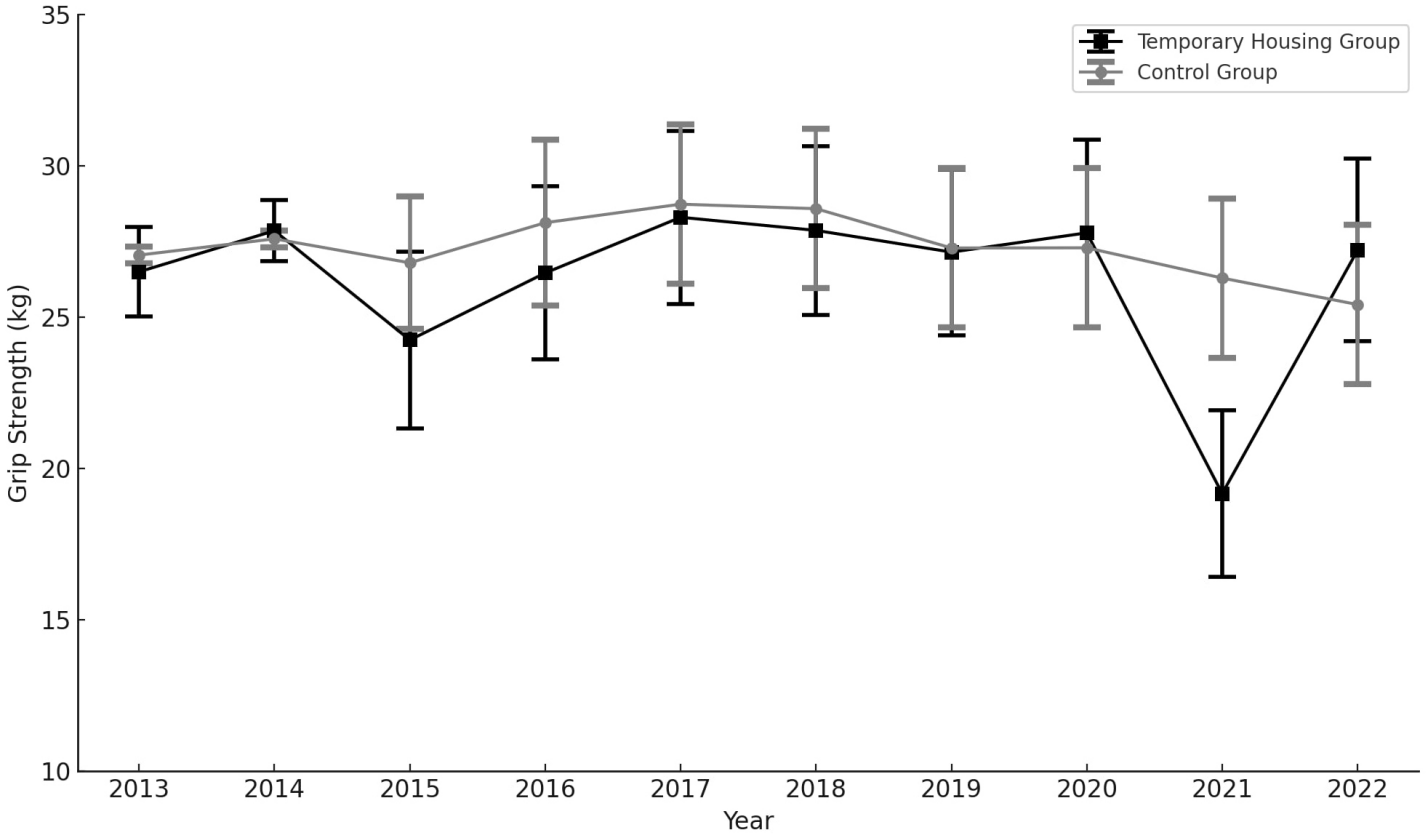
Sex- and age-adjusted changes in grip strength over 10 years. Figure 2 shows sex- and age-adjusted grip strength over 10 years. The solid black line represents the results for the control group, and the gray dashed line represents the results for the temporary housing (TH) group. The vertical axis represents grip strength, and the horizontal axis represents the year.

**Figure 3.**
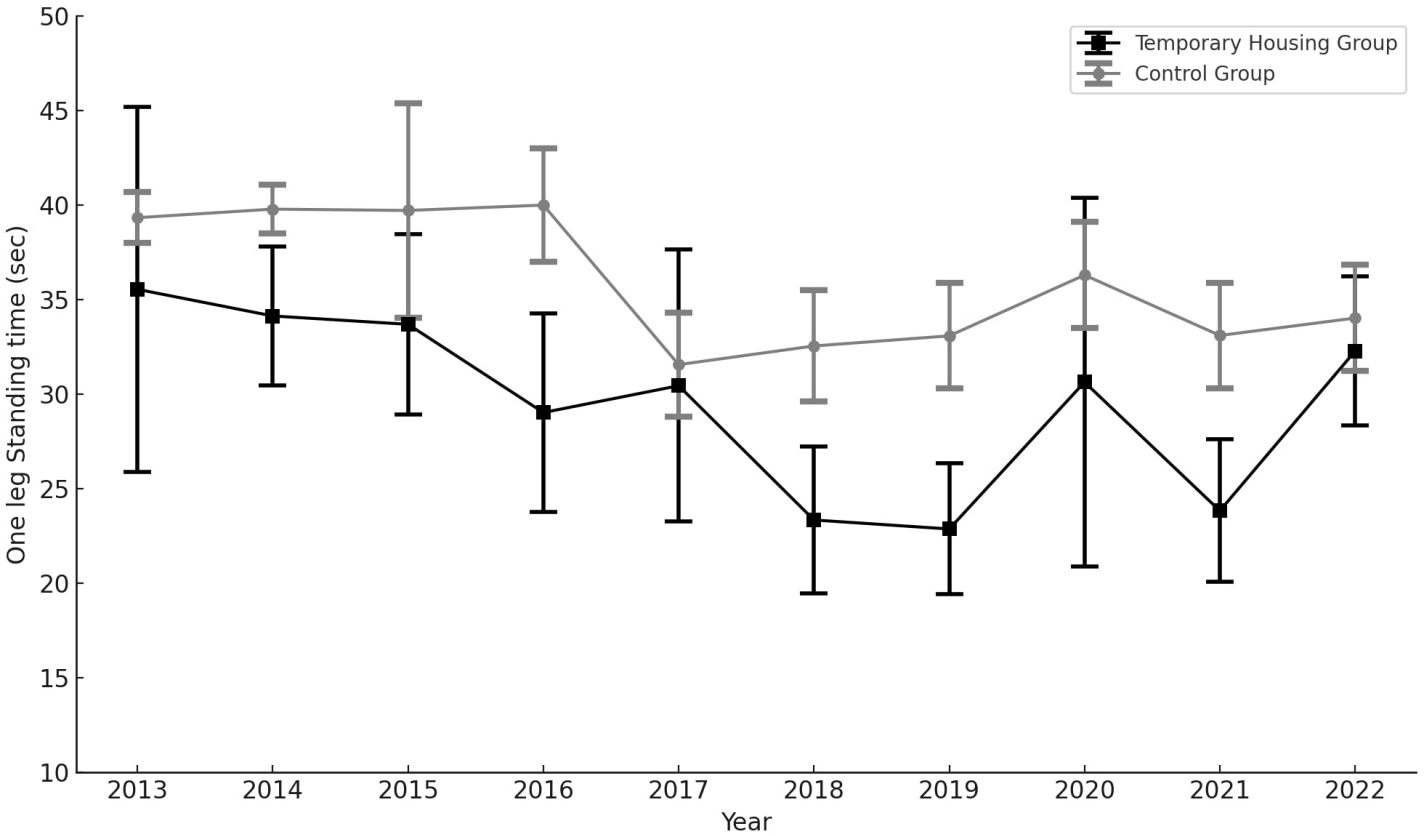
Sex- and age-adjusted changes in one-leg standing time over 10 years. Figure 3 shows sex- and age-adjusted one-leg standing time over 10 years. The solid black line represents the results for the control group, and the gray dashed line represents the results for the temporary housing (TH) group. The vertical axis represents one-leg standing time, and the horizontal axis represents the year.

Among the 4,810 initial participants, 130 were excluded due to missing age data over the 10 years, leaving 4,680 participants for analysis (440 in the TH group and 4,240 in the control group). Table 1 presents the background information of the participants for each year. Supplementary Table 2 shows the crude means of grip strength and OLST results over the 10 years. Figure 2 and 3 illustrate the sex- and age-adjusted grip strength and OLST over the 10 years, respectively. Grip strength showed no substantial difference between the TH and control groups in 2013, with mean values of 26.5 kg (95% confidence interval [CI]: 25.0-28.0 kg) and 27.1 kg (95% CI: 26.8-27.3 kg), respectively. By 2022, the control group showed a mean grip strength of 25.4 kg (95% CI: 22.8-28.0 kg), whereas the corresponding value in the TH group was 27.2 kg (95% CI: 24.2-30.2 kg). The mean OLST in 2013 was 39.3 seconds (95% CI: 38.0-40.7 seconds) in the control group and 35.5 seconds (95% CI: 25.9-45.2 seconds) in the TH group. The mean OLST in the TH group was consistently lower than that in the control group throughout the study period.

**Table 1.** Characteristics of the Study Sample by Year.

Characteristics	Total	2013	2014	2015	2016	2017	2018	2019	2020	2021	2022
Participants, n	4,680	1,592	1,907	524	533	1,635	738	1,194	1,189	1,273	1,285
Sex, n (%)											
Female	2,720 (58.1)	644 (40.5)	1,083 (56.8)	443 (84.5)	462 (86.7)	964 (60.0)	435 (58.9)	706 (59.1)	706 (59.4)	773 (60.7)	740 (57.6)
Male	1,960 (41.9)	948 (59.6)	824 (43.2)	81 (15.5)	71 (13.3)	671 (41.0)	303 (41.1)	488 (40.9)	483 (40.6)	500 (39.3)	545 (42.4)
Age, mean (standard deviation)	-	72.7 (6.2)	72.4 (6.4)	73.8 (7.1)	74.6 (6.3)	72.9 (6.1)	72.7 (6.0)	73.1 (6.0)	73.2 (5.9)	73.3 (6.1)	73.4 (6.1)
Temporary housing experience, n (%)	440 (9.4)	59 (3.7)	251 (13.2)	159 (30.3)	152 (28.5)	164 (10.0)	80 (10.8)	98 (8.2)	108 (9.1)	98 (7.7)	98 (7.6)
Number of missing Grip strength values, n (%)	-	1 (0.06)	0 (0)	0 (0)	5 (0.9)	6 (0.4)	1 (0.1)	2 (0.2)	4 (0.3)	3 (0.2)	0 (0)
Number of missing “One leg standing time” values, n (%)	-	23 (1.4)	9 (0.5)	21 (4.0)	23 (4.3)	15 (0.9)	10 (1.4)	15 (1.3)	15 (1.3)	11 (0.9)	16 (1.2)

Table 1 presents the total or mean value of each item in the survey sample by year.

### GCM of grip strength

Table 2 presents the estimated mean values and 95% CIs of the latent growth parameters (intercepts and slopes) for these variables. The results showed no differences in the intercept, slope, and slope 2 for “temporary housing experience.” For “sex (reference = female),” the intercept estimate was −11.48 (95% CI: −11.86 to −11.11), the slope was −0.54 (95% CI: −0.71 to −0.37), and the slope 2 was 0.06 (95% CI: 0.04-0.06), indicating a difference. “Age” also showed a difference, with an intercept estimate of −0.52 (95% CI: −0.54 to −0.50), a slope of 0.14 (95% CI: 0.13-0.14), and a slope 2 of −0.02 (95% CI: −0.02 to −0.02). The model fit indices for grip strength (CFI = 0.946, RMSEA = 0.052, AIC = 1120.8) met the recommended cut-off values (Supplementary Table 1).

**Table 2.** Estimated Means and 95% CIs for Outcomes by Age, Sex, and Evacuation Experience.

	Temporary housing experience		Sex (ref: female)		Age	
	Estimated value	95% CI	Estimated value	95% CI	Estimated value	95% CI
Outcome: grip						
Intercept	0.11	(−0.52, 0.74)	−11.48	(−11.86, −11.11)	−0.52	(−0.54, −0.50)
Slope	−0.27	(−0.56, 0.03)	−0.54	(−0.71, −0.37)	0.14	(0.13, 0.14)
Slope 2	0.02	(−0.01, 0.06)	0.06	(0.04, 0.06)	−0.02	(−0.02, −0.02)
Outcome: one-leg standing time						
Intercept	−4.32	(−7.49, −1.16)	0.86	(−1.00, 2.73)	−2.02	(−2.13, −1.91)
Slope	−0.05	(−1.43, 1.33)	−0.27	(−1.07, 0.54)	0.08	(0.06, 0.10)
Slope 2	0.03	(−0.12, 0.18)	0.03	(−0.06, 0.18)	−0.01	(−0.01, −0.01)

CI: confidence interval; Ref: reference. This table presents the results from the latent growth curve model, showing the estimated unstandardized regression coefficients and their 95% confidence intervals (CI) for grip strength and one-leg standing time outcomes. ICEPT: Initial level (intercept), representing baseline values in 2013 SLOPE: Linear change rate over time SLOPE2: Quadratic (accelerated) change over time

### GCM of OLST

The lower row of Table 2 shows an association for “temporary housing experience,” with an intercept estimate of −4.32 (95% CI: −7.49 to −1.16). Although the slope and slope 2 for “temporary housing experience” were not significantly different, they indicated a slower decline (−0.05) compared to the control group. No differences were found in the intercept, slope, or slope 2 for “sex (reference = female).” “Age” showed associations with the intercept (−2.02 [95% CI: −2.13 to −1.91]), slope (0.08 [95% CI: 0.06-0.10]) and slope 2 (−0.01 [95% CI: −0.01 to −0.01]). The model fit indices for OLST (CFI = 0.983, RMSEA = 0.018, AIC = 246.9) met the recommended cut-off values (Supplementary Table 1).

## Discussion

This study conducted a GCM to compare the long-term effects of living in TH on motor function in older adults in Soma City, which was severely affected by the Great East Japan Earthquake. The results indicated that older adults with experience living in TH had a shorter OLST throughout the analysis period compared to those without such experience. In contrast, grip strength did not differ between the two groups. These results suggest that the experience of living in TH had limited long-term effects on muscle weakness in older adults but had persistent negative effects on OLST, which reflects balance ability.

This study found that those who moved into TH after the earthquake experienced a long-term decline in OLST. A decline in OLST among TH residents was also observed in a previous cross-sectional study in the same area in 2012 ^(19)^. This study presents a new finding: OLST was shorter for elderly TH residents for an extended period after the earthquake compared to those without the experience of living in TH. Although OLST is primarily used as an indicator of physical balance ^(26), (27)^, it can also reflect multiple bodily functions ^(27), (33)^, such as attentional function, deep sensation, and neuromuscular control ^(34), (35)^, in addition to serving as a measure of muscle strength. These functions cannot be compensated for by strength training alone but can be activated by external stimuli, physical activity, and complex exercise in daily life ^(36)^.

Many individuals who experienced evacuation during the Great East Japan Earthquake were forced to change their jobs ^(37)^. In this region, where a significant portion of the population relies on primary industries such as fishing, the impact of job changes on residents in TH may have been particularly significant. Furthermore, a survey conducted 1 year after the move to TH revealed that 16.1% of residents reported a loss of social interactions ^(38)^. The combination of job changes, loss of social connections, and alterations in daily routines may have contributed to a decrease in physical activity among those in TH, resulting in a decline in OLST. In fact, the daily step count of residents in TH was lower than that of those who continued living in their own homes ^(39)^.

Notably, our study found that, compared to those who had not lived in TH, the OLST among older adults with TH experience did not improve after 2017, when TH operations ended. However, the findings of this study did not provide clear insights into the reasons for this persistent impact of living in TH. One possibility is that living in TH may have led to an increased incidence of illness, with the effects of these illnesses persisting over time. Cohort studies conducted after the Great East Japan Earthquake showed that individuals who experienced evacuation at least once had a higher risk of developing diabetes, obesity, and hypertension compared to those who did not experience evacuation ^(40), (41)^. Exacerbation of diabetes and kidney disease is known to negatively affect proprioception and superficial sensations, leading to a deterioration in OLST ^(42), (43)^. Further research is needed to clarify the relationship between living in TH, the development of illnesses, and long-term effects on balance ability.

The present study showed no difference in grip strength regardless of the experience of living in TH. This result, as well as the findings for the OLST, aligns with the results of a previous study in the same area in 2012 ^(19)^. Grip strength is a measure that primarily reflects overall muscle strength ^(11), (23)^ and has been incorporated into some assessment tools for frailty and sarcopenia ^(44)^. However, no previous study has clarified whether reductions in muscle strength occur throughout the course of evacuation after a disaster. Nevertheless, the risk of requiring long-term care increases after a disaster, and a combination of muscular and non-muscular factors can be expected to contribute to a decline in physical function.

If the experience of living in TH is likely to lead to a deterioration, specifically in balance ability, it is important to develop exercise interventions and health programs that not only improve muscle strength but also enhance balance and fall prevention among those who have experienced evacuation after a disaster. In Soma City, various initiatives have been reported, including the establishment of a social community to prevent the decline in physical function and the increased need for care due to evacuation ^(45), (46)^, as well as physical activity improvement programs and exercise classes targeting evacuees ^(47), (48)^. These initiatives may have contributed, at least in part, to the maintenance of muscle strength among residents. Although the effectiveness of these interventions could not be evaluated in the present study, our results may reflect the impact of programs aimed at improving physical function among evacuees living in TH in the Soma region. The effectiveness of these initiatives should be evaluated to support preparedness for future disasters.

### Implications

The results of the current study suggest that an appropriate support strategy for evacuees who have moved into TH should include comprehensive physical exercises for improving balance capacity, trunk stability, and attention. According to a previous study that focused on the physical activity environment and balance capacity, effective training for improving balance capacity includes a combination of coordination training, physical activity, and strength training ^(49)^. Furthermore, interventions that promote social activities and community engagement may be beneficial in improving OLST even after the evacuation has ended. A study targeting community-dwelling older adults aged 70-84 years in South Korea indicated an association between limited social interactions and pre-frailty. In post-disaster situations, fostering social interactions is likely to be effective in preventing a decline in overall physical function ^(50)^. One direction for future research would be to investigate whether evacuees who have experienced a long-term decline in balance ability are at increased risk of illnesses, such as fractures and falls, as well as changes in their level of independence. In particular, developing and implementing comprehensive support measures from both long-term and short-term perspectives is important. Furthermore, future studies should aim to delve deeper into the connections between individuals’ backgrounds and environments to identify the specific factors contributing to their decline.

### Strengths and limitations

The strength of this study lies in its detailed analysis of the changes in physical functions among older adults living in TH. This allowed us to clearly demonstrate the long-term effects of disasters on physical function. However, the study had several limitations. First, important details regarding individual background characteristics―such as complications, living environment, duration of time spent in TH, activity level, nutritional status, and job status―were missing. Second, because the study involved participant screening, selection bias cannot be ruled out. However, it should be noted that the affected participants lost their homes due to the tsunami and nuclear accident, not because they were already facing socioeconomic disadvantages. TH was allocated to those who lost their homes in the disaster, regardless of their medical history or income. Although our adjustments for confounding factors may have been insufficient, we believe there were no substantial differences between the two groups in attributes that could affect outcomes, such as chronic diseases or social disparities. Third, since the study included participants who had undergone evacuation, it may have overestimated the trend in motor function within the population. In the context of an unprecedented disaster such as the Fukushima Daiichi Nuclear Power Station accident, considering the immediate needs and vulnerabilities of the affected population, conducting interventions with research as the primary objective is often considered unacceptable and met with aversion by residents. To protect the long-term health of affected populations in future disasters, research should include a detailed assessment of individual circumstances and evaluate the effectiveness of supportive measures based on these assessments. In addition, future studies should assess residents’ individual circumstances in detail and evaluate the effectiveness of support measures based on these assessments.

### Conclusions

Moving into TH had a negative medium- to long-term effect on OLST. In contrast, grip strength―an indicator of overall muscle strength―was not affected by the TH relocation. A Decline in balance function is a major factor that increases the risk of falls, leading to the need for nursing care and frailty. Therefore, residents moving into TH after a disaster should be aware of the physical risks, and efforts should be made to provide multifaceted exercise interventions and physical function improvements from an early stage.

## Article Information

### Conflicts of Interest

None

### Sources of Funding

The author(s) declare that financial support was received for the research, authorship, and/or publication of this article. This work was supported by the Research Project on the Health Effects of Radiation organized by the Ministry of Environment, Japan. This work was supported by the Program of the Network-type Joint Usage/Research Center for Radiation Disaster Medical Science.

### Acknowledgement

We would like to thank all staff from Houeikai, a medical corporation, who contributed to data acquisition and management. We would like to thank Dr. Hiroyuki Yonemura and Dr. Hiroshi Funabashi from the Soma Medical Association, Soma City, for their support with data acquisition and management. We would also like to take this opportunity to thank the staff at the Soma City Health Center and City Hall, the many supporters of the health checkups, and Ms. Michiko Watanabe of the Department of Radiation Health Management. We would like to thank Editage (www.editage.jp) for English language editing.

### Author Contributions

Toshiki Abe, Hiroaki Saito, and Nobuaki Moriyama conceived and designed the study. Toshiki Abe, Hiroaki Saito, Nobuaki Moriyama, and Michio Murakami collected and analyzed the data. Toshiki Abe and Hiroaki Saito wrote the paper. Naomi Ito, Yoshitaka Nishikawa, Morihito Takita, Isamu Amir, Yoshitaka Shiba, Takeaki Ishii, Sae Ochi, Chika Yamamoto, Tianchen Zhao, Makoto Kosaka, and Masaharu Tsubokura supervised the study and provided critical comments on the manuscript. All authors approved the final version of the manuscript and agreed to be accountable for all aspects of this work.

### Approval by Institutional Review Board (IRB)

IRB approval code issued: REC2024-059. The name of the institution that granted approval: The Fukushima Medical University Ethics Committee.

## Supplement

Supplementary Figure 1. Diagram of the latent growth curve model
Supplementary Figure 1 shows a linear latent growth curve model that analyzes the associations among the factors “age,” “sex,” and “temporary housing experience,” and their influence on grip strength and one-leg standing time (OLST) results over 10 years period (2013-2022). X represents either grip strength or one-leg standing time, both of which were incorporated into the model. The values shown are unstandardized path coefficients. The linear models

Supplementary Tables Supplementary Table 1. Comparative Fit Index (CFI), Root Mean Square Error of Approximation (RMSEA), and Akaike Information Criterion (AIC) for Each Growth Curve Model.
Supplementary Table 1 presents the goodness-of-fit indices for each growth curve model regarding the age variable. Among the four models, the continuous quadratic form of age demonstrated the highest fit and was therefore adopted.
Supplementary Table 2. Crude means of grip strength and one-leg standing time values over 10 years
Supplementary Table 2 shows the mean grip strength and one-leg standing time data for each group in the survey sample by year.
