# Supplementary figures and images for: Physical Function Changes in Older Adults Living in Temporary Housing after the Great East Japan Earthquake
Source: JMA J. 2025 Jul 2;8(3):779–88. doi: 10.31662/jmaj.2025-0121 (PMC12328901; doi:10.31662/jmaj.2025-0121)

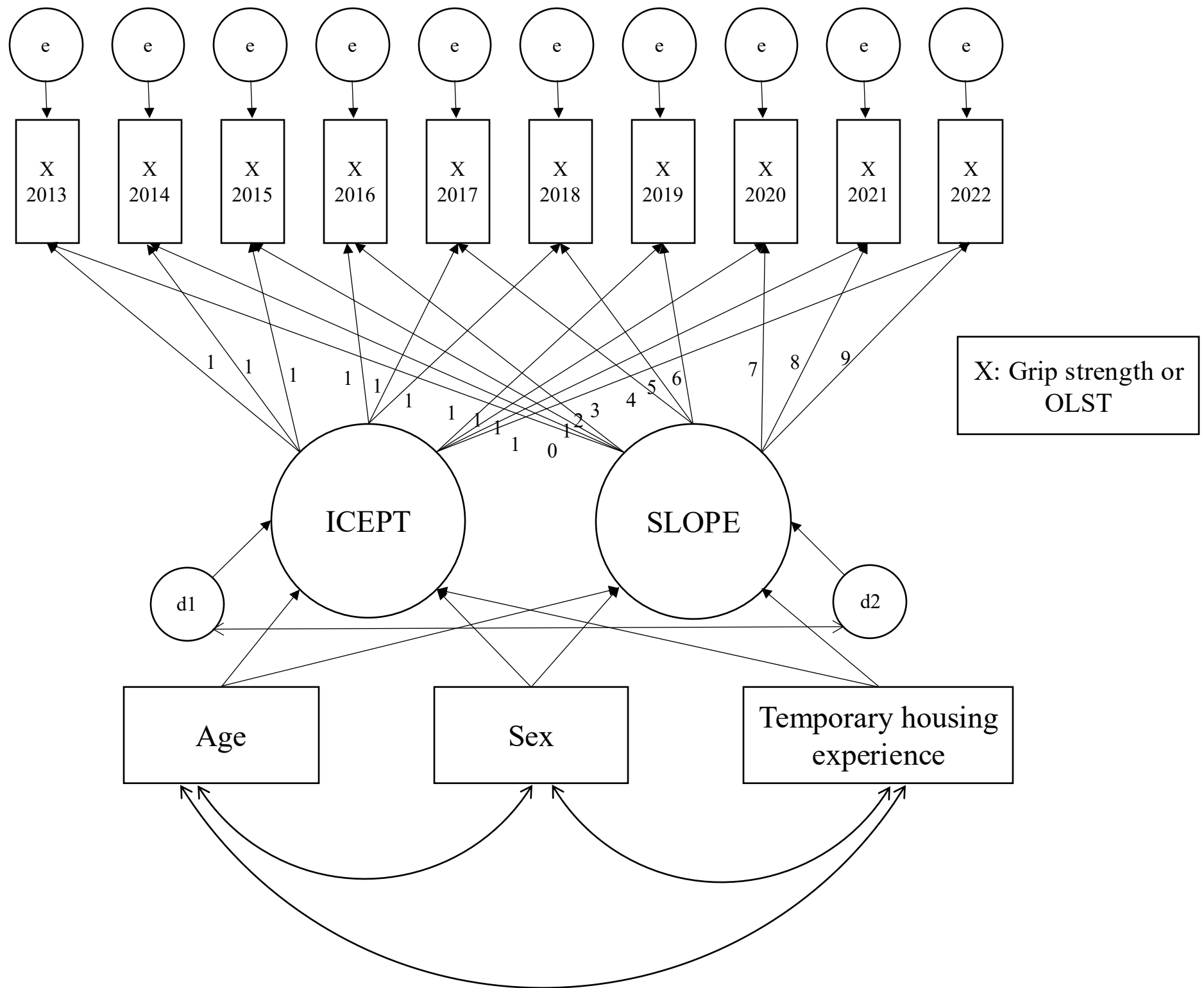

Supplement: Supplementary Figure 1. — Diagram of the latent growth curve model Supplementary Figure 1 shows a linear latent growth curve model that analyzes the associations among the factors “age,” “sex,” and “temporary housing experience,” and their influence on grip strength and one-leg standing time (OLST) results over 10 years period (2013-2022). X represents either grip strength or one-leg standing time, both of which were incorporated into the model. The values shown are unstandardized path coefficients. The linear models [file 2433-3298-8-3-0779-s001.pdf]
