# Supplementary material for: Physical Function Changes in Older Adults Living in Temporary Housing after the Great East Japan Earthquake
Source: JMA J. 2025 Jul 2;8(3):779–88. doi: 10.31662/jmaj.2025-0121 (PMC12328901; doi:10.31662/jmaj.2025-0121)
Supplement: Supplementary Tables — Supplementary Table 1. Comparative Fit Index (CFI), Root Mean Square Error of Approximation (RMSEA), and Akaike Information Criterion (AIC) for Each Growth Curve Model. Supplementary Table 1 presents the goodness-of-fit indices for each growth curve model regarding the age variable. Among the four models, the continuous quadratic form of age demonstrated the highest fit and was therefore adopted. Supplementary Table 2. Crude means of grip strength and one-leg standing time values over 10 years Supplementary Table 2 shows the mean grip strength and one-leg standing time data for each group in the survey sample by year. [file 2433-3298-8-3-0779-s002.pdf]

Supplementary Table 1. Comparative Fit Index (CFI), Root Mean Square Error of Approximation (RMSEA), and Akaike Information Criterion (AIC) for each growth curve model

Model	Age	Linear/Quadratic	CFI	RMSEA	AIC
Grip Strength					
GCM1_G	Continuous variable	Linear	0.941	0.053	1216.4
GCM2_G	Categorized: 65–74 years and ≥75 years	Linear	0.938	0.053	1208.7
GCM3_G	Continuous variable	Quadratic	**0.946**	**0.052**	**1120.8**
GCM4_G	Categorized: 65–74 years and ≥75 years	Quadratic	0.677	0.126	5818.2
One-leg Standing Time					
GCM1_O	Continuous variable	Linear	0.977	0.021	289.0
GCM2_O	Categorized: 65–74 years and ≥75 years	Linear	0.972	0.021	301.5
GCM3_O	Continuous variable	Quadratic	**0.983**	**0.018**	**246.9**
GCM4_O	Categorized: 65–74 years and ≥75 years	Quadratic	0.941	0.032	496.2

Supplementary Table 2. Crude means of grip strength and one-leg standing time values over the 10-year period

Assessment Items	2013	2014	2015	2016	2017	2018	2019	2020	2021	2022
Mean (standard deviation) of grip strength, kg										
Control group	26.5 (7. 5)	27.9 (7.8)	24.1 (5.7)	22.9 (6.1)	29.5 (8.1)	29.8 (8.5)	28.7 (8.8)	29.0 (8.7)	28.3 (8.0)	28.3 (8.1)
TH group*	26.6 (6.8)	28.3 (8.8)	25.7 (8.7)	25.6 (8.3)	29.0 (8.1)	28.2 (8.0)	29.1 (8.2)	28. 9 (8.1)	28.8 (7.7)	28.1 (8.0)
Total	26.5 (7.5)	28.0 (8.0)	24.6 (6.7)	23.7 (6.9)	29.5 (8.1)	29.6 (8.5)	28.8 (8.7)	29.0 (8.6)	28.4 (8.0)	28.2 (8.2)
Mean (standard deviation) of one leg standing time, s										
Control group	42.5 (27.8)	44.7 (27.4)	44.3 (26.6)	39.5 (26.7)	41.9 (27.3)	43.0 (27.7)	44.9 (27.2)	48.7 (26.2)	48.2 (27.4)	49.3 (26.2)
TH group	40.7 (26.9)	40.8 (27.4)	40.7 (25.2)	35.7 (25.1)	40.9 (27.6)	38.3 (28.6)	39.6 (25.2)	46.8 (26.6)	48.9 (27.7)	52.0 (27.0)
Total	42.4 (27.8)	44.2 (27.4)	43.2 (26.2)	38.4 (26.3)	41.8 (27.3)	42.4 (27.8)	44.5 (27.1)	48.5 (26.3)	48.3 (27.4)	49.5 (26.2)

* TH group: temporary housing experience group
